# Safety and Efficacy of Myval Implantation in Patients with Severe Bicuspid Aortic Valve Stenosis—A Multicenter Real-World Experience

**DOI:** 10.3390/jcm11020443

**Published:** 2022-01-15

**Authors:** Ahmed Elkoumy, John Jose, Christian J. Terkelsen, Henrik Nissen, Sengottuvelu Gunasekaran, Mahmoud Abdelshafy, Ashok Seth, Hesham Elzomor, Sreenivas Kumar, Francesco Bedogni, Alfonso Ielasi, Santosh K. Dora, Sharad Chandra, Keyur Parikh, Daniel Unic, William Wijns, Andreas Baumbach, Darren Mylotte, Patrick Serruys, Osama Soliman

**Affiliations:** 1 Discipline of Cardiology, Saolta Group, Galway University Hospital, Health Service Executive and CORRIB Core Lab, National University of Ireland Galway (NUIG), H91 V4AY Galway, Ireland; Ahmed.elkoumy@nuigalway.ie (A.E.); Mahmoud.Abdelshafy@nuigalway.ie (M.A.); Hesham.Elzomor@nuigalway.ie (H.E.); william.wyns@gmail.com (W.W.); darrenmylotte@gmail.com (D.M.); 2 Department of Cardiology, Christian Medical College & Hospital, Vellore 632004, India; drjohnjose@gmail.com; 3 Department of Cardiology, Aarhus University Hospital, DK-8200 Aarhus, Denmark; christian.terkelsen@skejby.rm.dk; 4 Department of Cardiology, Odense University Hospital, DK-5000 Odense, Denmark; henrik.nissen@rsyd.dk; 5 Department of Cardiology, Apollo Main Hospital, Greams Road, Chennai 600006, India; drgseng@gmail.com; 6 Fortis Escorts Heart Institute, New Delhi 110025, India; ashok.seth@fortishealthcare.com; 7 Department of Cardiology, Apollo Hospitals, Apollo Health City, Jubilee Hills, Hyderabad 500050, India; arramraj@yahoo.com; 8 Department of Cardiology, IRCCS Policlinico San Donato, 20097 Milan, Italy; francesco.bedogni@grupposandonato.it; 9 Clinical and Interventional Cardiology Unit, Istituto Clinico Sant’Ambrogio, 20149 Milan, Italy; alielasi@hotmail.com; 10 Asian Heart Institute, Mumbai 00051, India; santosh.dora@ahirc.com; 11 Department of Cardiology, King George’s Medical University, Lucknow 226003, India; sharadaiims@gmail.com; 12 Care Institute of Medical Sciences, Ahmedabad 380060, India; keyur.parikh@cims.org; 13 Department of Cardiac and Transplant Surgery, University Hospital Dubrava, 10000 Zagreb, Croatia; danielunic75@gmail.com; 14 CÚRAM, The SFI Research Centre for Medical Devices, H91 TK33 Galway, Ireland; 15 William Harvey Research Institute, Queen Mary University of London and Barts Heart Centre, London EC1M 6BQ, UK; a.baumbach@qmul.ac.uk; 16 National Heart and Lung Institute (NHLI), Imperial College London, London SW7 2AZ, UK

**Keywords:** aortic stenosis, bicuspid aortic valve, transcatheter aortic valve implantation

## Abstract

Bicuspid aortic valve (BAV) is the most common valvular congenital anomaly and is apparent in nearly 50% of candidates for AV replacement. While transcatheter aortic valve implantation (TAVI) is a recommended treatment for patients with symptomatic severe aortic stenosis (AS) at all surgical risk levels, experience with TAVI in severe bicuspid AS is limited. TAVI in BAV is still a challenge due to its association with multiple and complex anatomical considerations. A retrospective study has been conducted to investigate TAVI’s procedural and 30-day outcomes using the Myval transcatheter heart valve (THV) (Meril Life Sciences Pvt. Ltd. Vapi, Gujarat, India) in patients with severe bicuspid AS. Data were collected on 68 patients with severe bicuspid AS who underwent TAVI with the Myval THV. Baseline characteristics, procedural, 30-day echocardiographic and clinical outcomes were collected. The mean age and STS PROM score were 72.6 ± 9.4 and 3.54 ± 2.1. Procedures were performed via the transfemoral route in 98.5%. Major vascular complications (1.5%) and life-threatening bleeding (1.5%) occurred infrequently. No patient had coronary obstruction, second valve implantation or conversion to surgery. On 30-day echocardiography, the mean transvalvular gradient and effective orifice area were 9.8 ± 4.5 mmHg and 1.8 ± 0.4 cm^2^, respectively. None/trace aortic regurgitation occurred in 76.5%, mild AR in 20.5% and moderate AR in 3%. The permanent pacemaker implantation rate was 8.5% and 30-day all-cause death occurred in 3.0% of cases. TAVI with the Myval THV in selected BAV anatomy is associated with favorable short-term hemodynamic and clinical outcomes.

## 1. Introduction

Bicuspid aortic valve (BAV) is the most common valvular congenital anomaly occurring in up to 1.5% of the general population [1]. In addition, approximately 50% of patients with aortic valve (AV) dysfunction and indicated for aortic valve replacement (AVR) have BAV anatomy [2]. Furthermore, BAV is the predominant cause of severe aortic stenosis (AS) in patients ≤70 years [2,3].

The recent ACC/AHA and ESC guidelines have recommended transcatheter aortic valve implantation (TAVI) as a viable and alternative treatment to surgical aortic valve replacement (SAVR) for selected patients with severe AS. Importantly, neither guideline included patients with BAV in their specific recommendations [4,5].

BAV is a heterogenous valvulo-aortopathy disorder associated with valve leaflet dysfunction and/or aortic dilatation [6]. Anatomic heterogeneity of BAV arises from the eccentricity of valve opening, different leaflet and raphe phenotypes (Sievers’ classification; type 0, type-1 and type-2 [7]), asymmetry in the sinus of Valsalva and variable degrees and localization of calcification (leaflet, raphe, annulus and LVOT) [8].

Although BAV has been considered as unfavorable valve anatomy for TAVI [4,5] and therefore was excluded from all completed randomized controlled trials (RCTs) [4,5], several cohort studies and registries have been conducted to test TAVI feasibility in BAV among different surgical risk groups, using various transcatheter heart valves (THV).

The selection of the most appropriate THV device and size is challenging in BAV due to the unpredictable baseline anatomy. In a recent paper by Kawashima et al. [9], operators from real-world practice opted for intermediate size selection in approximately half of the patients treated with TAVI using the BEV Myval (Meril Life Sciences Pvt. Ltd., India). The Myval intermediate sizes are designed with a 1.5 mm increment instead of the conventional 3 mm increment of sizes in most of the commercially available TAVI devices. TAVI using the Myval in tricuspid AV shows favorable results [10,11,12]. Among other features, the availability of additional sizes may furnish the Myval system with unique advantages in treating BAV morphology. Hence, we sought to investigate the procedural outcomes in addition to 30-day safety and performance of TAVI using the Myval THV in patients with severe bicuspid AS.

## 2. Materials and Methods

Data of patients confirmed to have BAV and selected for TAVI were collected retrospectively from 12 centers in India (*n* = 7), Denmark (*n* = 2), Italy (*n* = 2), and Croatia (*n* = 1). A list of participating centers, collaborators and numbers of patients included is provided in Supplementary Table S2. Patients were treated between 2018 and 2021 with the Myval THV. The decision to perform TAVI was made by the local heart team in each center, in adherence to the recent guidelines’ recommendations, and none of the patients had a clear surgical indication such as dilated aorta as defined by the ACC/AHA or ESC guidelines in patients with BAV [4,13]. Informed consent was obtained from each participant for TAVI. The Navigator-THV delivery system and Python 14-F expandable introducer sheath (Meril Life Sciences Pvt. Ltd., India) were used Procedures were performed according to local clinical practice guidelines.

Evaluation of the aortic valve morphology was performed using TTE during the initial echocardiographic assessment and confirmed by MSCT during the pre-TAVI assessment.

Preprocedural MSCT analysis was performed either at the participating centers (*n* = 5) or a CT Core Lab (Meril Life Sciences Pvt. Ltd., India). Clinical and procedural data, in addition to 30-day clinical outcomes, including site-reported echocardiographic assessment reported by the participating centers, were collected by the CORRIB Core Laboratory (National University of Ireland Galway, Galway, Ireland). This registry was conducted as an academic collaboration among the participating centers without sponsoring from the industry.

1. Endpoints and definitions

Technical success, device success and early safety endpoints were adjudicated according to the updated Valve Academic Research Consortium (VARC-3) [14]. The left ventricular (LV) systolic function and device hemodynamic assessments including the severity of paravalvular aortic regurgitation (AR) were assessed and graded using transthoracic echocardiography (TTE) according to established guidelines [14,15,16,17,18]. Aortic regurgitation was reported as total AR and was classified as none/trace, mild, moderate, or severe.

2. Statistical analysis

Continuous variables were presented as mean and standard deviation (SD) after conduction of the Shapiro–Wilk test for normality. Categorical variables were presented as frequencies and percentages. Analysis was performed using the IBM^®^ SPSS^®^ Statistics version 27 (IBM Corp. in Armonk, NY, USA).

## 3. Results

### 3.1. Study Population and Baseline Characteristics

**Figure 1:**
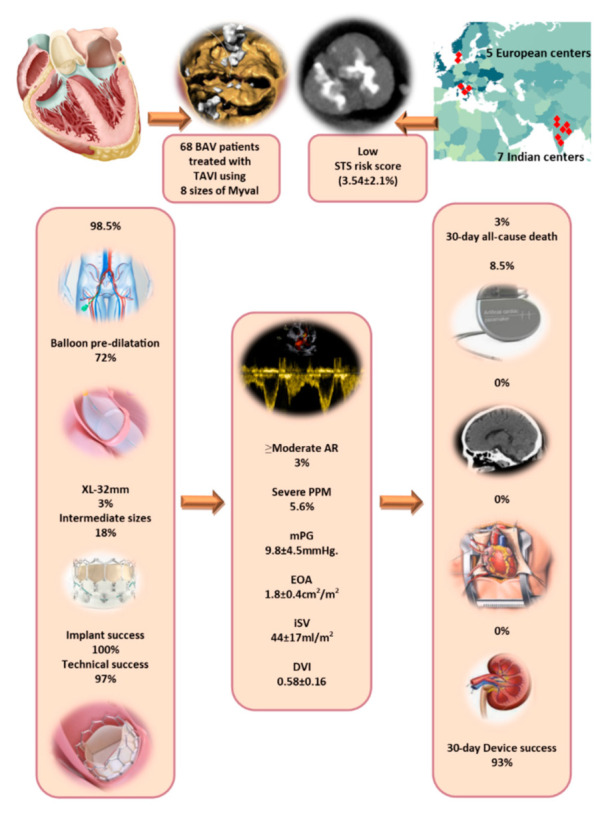
Visual abstract of the Myval bicuspid registry summarizing the key findings of the registry; including the procedural details and outcome, 30-day echocardiographic and clinical outcomes. AR: Aortic regurgitation; iSV: indexed stroke volume; mPG: mean pressure gradient; PPM: patient prosthesis mismatch; DVI: dimensionless velocity index. EOA: Effecive orifice area; BAV: Bicuspid aortic valve STS: Society of Thoracic Surgeons; TAVI: Transcatheter aortic valve implanttaion.

**Figure 2:**
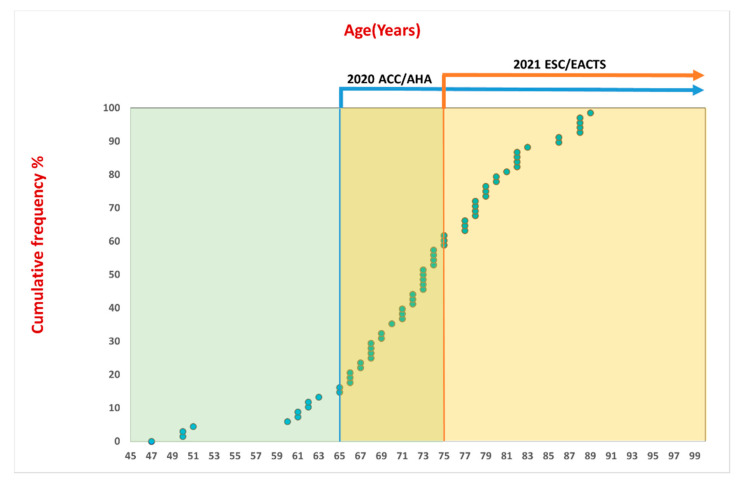
Cumulative curve showing the age groups of the included patients in the registry. The figure shows the cut-off age recommended by the ACC/AHA, 2020 and ESC/EACTS 2021 guidelines.

The study comprised 68 patients with severe bicuspid AS who underwent TAVI using the Myval THV system. Patients’ mean age was 72.6 ± 9.4 years, (Figure 1 and Figure 2) and most (72%) were male. The BAV type was reported in 62 (91%) patients, and according to the Sievers’ classification 12 (19%) patients were type 0, 46 (74%) patients were type 1, and 4 (7%) were type 2. The average Society of Thoracic Surgeons (STS) Predicted Risk of Mortality score was 3.5 ± 2.1%. Patients baseline characteristics are summarized in Table 1.

### 3.2. Procedural Characteristics

TAVI was performed through femoral access in 67 (98.5%) patients. The Python 14-F expandable introducer sheath was used in 67 (98.5%) patients. TAVI was performed with local anesthesia and/or moderate sedation in all cases. Preimplantation balloon dilatation was used in 49 (72%) patients and postimplantation was performed only in 8 (12%) patients (Table 2). Eight different sizes of Myval were used including the novel extra-large devices (32 mm-XL) which were implanted in two patients, while, in 12 (18%) cases the intermediate device sizes were required (Figure 1).

### 3.3. Clinical Outcomes

VARC-3 technical success was achieved in 97% of patients. No procedural death and no need for second valve implantation or conversion to surgery were observed. Life-threatening bleeding occurred in one (1.5%) patient and an access-related complication occurred in another single (1.5%) case (Table 3). VARC-3 device success at 30 days was achieved in 93% (Table 4). Two (3.0%) deaths were reported at 30 days: one patient had baseline untreated coronary artery disease and developed acute myocardial infarction and the other patient had sudden cardiac death. New permanent pacemaker implantation was necessary for six (8.5%) patients. There were no disabling strokes, no acute kidney injury, and no cases required reintervention related to the device (neither surgery nor transcatheter). Early safety at 30 days was achieved in 91% (Table 4) (Figure 1).

### 3.4. Echocardiographic Outcome

**Figure 3:**
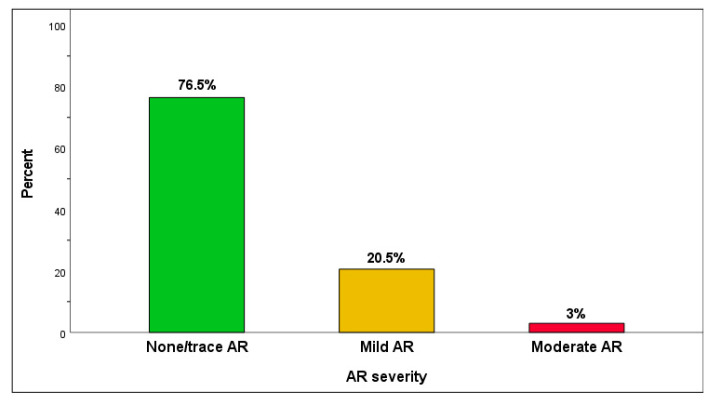
The frequency of aortic regurgitation (AR) severity was assessed with echocardiography at 30 days.

At 30 days, the echocardiographic data were as expected after TAVI using BEV (Table 5): mean transvalvular maximum velocity of 1.9 ± 0.5 m/s; mean pressure gradient of 9.8 ± 4.5 mmHg, effective orifice area of 1.8 ± 0.4 cm^2^. The total aortic regurgitation (AR) post-TAVI was non/trace AR in 52 (76.5%), mild AR in 14 (20.5%), moderate in 2 (3.0%), and there were no cases of severe AR (Figure 3). Severe prosthesis patient mismatch (PPM) was observed in three (5.6%) patients (Table 5).

## 4. Discussion

This is the first real-world experience of TAVI using the balloon-expandable Myval THV in patients with severe bicuspid AS. According to the VARC-3 definitions, the Myval system demonstrated favorable procedural, clinical, and hemodynamic outcomes at 30 days.

In the current study, the mean age was young (72.6 ± 9.4 years), with low surgical risk (STS PROM score 3.5 ± 2.1%), reflecting the typical prevalence of BAV. There was 3% mortality in the first 30 days and no disabling stroke. The device success rate was achieved in 93% of patients, with no cases of device migration, embolization, aortic root injury or need for second valve implantation. The low rate of significant post-TAVI AR in this registry is similar to the Myval performance in patients with severe AS and tricuspid anatomy [10,11,19].

The selection of surgical or transcatheter intervention in patients with severe bicuspid AS represents an emerging clinical challenge. On one hand, many patients would prefer to opt for TAVI, however no prospective randomized data have compared this approach to SAVR, which has well-established evidence in such groups. Recent guidelines suggest that SAVR should be the treatment of choice in younger and lower-risk patients with severe bicuspid AS [4,5], but this is more ambiguous in older or intermediate-to-high-risk candidates. Such recommendations are based on the exclusion of BAV patients from all completed RCTs and because there are legitimate reasons that BAV disease could yield inferior outcomes in BAV compared to tricuspid anatomy. The high prevalence of BAV in the younger population aged 50–70 years raises issues such as the occurrence of acute complications such as stroke and PMI and their impact on quality of life, THV durability with longer life expectancy and ultimately the need for valve reintervention in the future [20]. Interestingly, the US guidelines restrict TAVI use to patients ≥65 years [5] and European guidelines raise the bar for TAVI use only in patients ≥75 years [4].

BAV anatomy is challenging for TAVI due to valve-opening asymmetry, the fused raphe, differential sinuses’ depths and dimensions, significant calcifications, and associated aortopathy, in addition to the likely associated abnormal coronary origin. These anatomic constraints can potentially result in an asymmetric expansion of the implanted device, resulting in the requirement for aggressive postdilatation to treat residual gradients or paravalvular leak. Furthermore, the long-term mal expansion can lead to flow abnormalities, with the potential for device thrombosis and early structural valve deterioration, which ultimately may affect device durability [21,22].

The calcification severity is another major challenge for TAVI in bicuspid anatomy. Yoon et al. studied the two-year outcome of TAVI in low-surgical-risk patients with BAV. The authors reported a 4-fold increase in mortality in patients who had severe calcifications involving both leaflets and raphe, compared to patients with only leaflet calcification [23].

The experience of TAVI in BAV with the BEV, SEV, and mechanical expandable valves showed various outcomes, particularly in mortality, stroke, the need for permanent PMI, and AR severity [3].

The clinical outcomes of TAVI using the Myval BEV in BAV in this small registry are similar to those reported from contemporary BEV and SEV by Yoon et al. [23] with respect to all-cause mortality (3% vs. 2%), stroke (0% vs. 2.7%), and PMI (8.5% vs. 12.2%) at 30 days. Likewise, our results are comparable to the recently published data by Makkar et al. [24] regarding the TAVI outcome in low-surgical-risk BAV patients using contemporary BEVs. The need for new PMI (8.5% vs. 8.1%) and all-cause stroke (0% vs. 1.4%) [24].

Furthermore, the hemodynamic performance parameters of the Myval BEV are also comparable to those reported by Yoon et al. and by Makkar et al.—EOA is 1.8 ± 0.4 vs. 1.7 ± 0.5 and 1.9 ± 0.6, respectively, the mean pressure gradient is 9.8 ± 4.5 vs. 10.6 ± 5.0 and 12.7 ± 5.4 mmHg, respectively, and there was moderate or more AR (3% vs. 3.2% and 0.9%, respectively). Regarding the recent guidelines’ definition [14,16], PPM appeared as severe PPM at 13.8% in Makkar et al. in comparison to 5.6% (three patients) in our study (Table 5).

Life-threatening bleeding was seen only in one (1.5%) patient in the form of severe pericardial effusion (tamponade), and it was due to injury from the pacing lead, but the patient become stable after pericardiocentesis and did not require any reintervention.

To date, the postprocedural complications of TAVI in bicuspid AS using different prostheses have been low in several series [23,24,25]. These findings might, however, suggest a selection bias towards favorable valve anatomy and lower-risk patients. Indeed, the high rate of technical and device success, as well as early safety of TAVI using the Myval in this registry, could be explained by patient selection and proper sizing methodology in analyzing the BAV anatomy [26,27,28]. The use of novel intermediate sizes of the Myval technology could be beneficial in such difficult anatomy to avoid significant over or under-sizing of the selected THV. The incremental value of the intermediate sizes in BAV requires further investigation.

Without a dedicated RCT exploring the safety and efficacy of TAVI in BAV anatomy, current evidence remains “hypothesis-generating”. However, there is accumulating evidence that TAVI in BAV using selected and appropriately sized devices in a selected population is safe [3].

### Limitations

Limitations of this registry include its retrospective nature. In addition, detailed anatomical characteristics (BAV phenotype, calcium volume and distribution) were not collected systematically. Additionally, the data were site-reported with the unavoidable variability of reports between sites in absence of an independent Core Lab analysis and independent clinical event adjudication. The missing data in this registry are listed in Supplementary Table S1. The relatively small number of included patients, when compared to the results of studies with a large population number, is also a relevant limitation. However, the results of initial experience using Myval in BAV are encouraging for safety and performance among selected populations and different geographic regions.

## 5. Conclusions

TAVI using the balloon-expandable Myval THV was associated with favorable short-term hemodynamic and clinical outcomes in selected patients with severe bicuspid AS. These findings should be confirmed in a prospective, randomized controlled and adequately powered study.

## Figures and Tables

**Table 1:** Patients’ demographic and clinical characteristics of the patients included in the Myval bicuspid registry.

Demographic Characteristics	
Age (Range)	72.6 ± 9.4 (47–80)
Men	49 (72%)
Women	19 (28%)
BSA m^2^	1.75 ± 0.24
BMI kg/m^2^	26.8 ± 6.5
BMI < 30 kg/cm^2^	47 (69%)
BMI ≥ 30 kg/cm^2^	21 (31%)
BAV Phenotype (Sievers’ classification)	62 (91%)
Sievers’ type 0	12 (19%)
Sievers’ type 1	46 (74%)
Sievers’ type 2	4 (7%)
Clinical characteristics	
STS risk score%	3.54 ± 2.1
NYHA class III/IV	46 (68%)
NYHA class I/II	22 (32%)
Prior atrial fibrillation	12 (18%)
Peripheral vascular disease	9 (13%)

Data are presented as mean ± SD or number (*n*) and frequency (%). BSA: Body surface area; BMI: body mass index; NYHA: New York Heart Association.

**Table 2:** Procedural characteristics of TAVI using Myval THV in patients with BAV.

Characteristic	*n* (%)
Access site	
Transfemoral	67 (98.5%)
Other	1 (1.5%)
Local anaesthesia and moderate sedation	68 (100%)
Balloon predilatation	49 (72%)
Balloon postdilatation	8 (12%)
Implanted Myval THV size	
20 mm	7 (10%)
21.5 mm	2 (3%)
23 mm	20 (29.4%)
24.5 mm	5 (7.4%)
26 mm	14 (20.6)
27.5 mm	5 (7.4%)
29 mm	13 (19%)
32 mm	2 (3%)
Python 14-F introducer sheath	67 (98.5%)
Another introducer sheath (22-F)	1 (1.5%)
Navigator THV delivery system	68 (100%)

**Table 3:** Procedural outcome (Technical success (at the exit from procedure room)) of the Myval THV in BAV.

Outcome	*n* (%)
Procedural death	0
Failed delivery	0
Major vascular complications	1 (1.5%)
Life-threatening bleeding	1 (1.5%)
Conversion to surgery	0
Device migration or embolization	0
Aortic root injury	0
Coronary obstruction	0
Successful positioning and implantation	68 (100%)

**Table 4:** 30 days hemodynamic and clinical outcomes (device success at 30-day) of the Myval THV in BAV.

Outcome	*n* (%)
Transvalvular maximum velocity >3 m/s	1 (1.5%)
Transvalvular mean pressure gradient ≥20 mmHg	1 (1.5%)
Dimensionless velocity index <0.25	0
Post-TAVI AR moderate or severe AR	2 (3%)
Early safety (outcome)	
All-cause mortality	2 (3%)
All stroke	0
Acute kidney injury (stage III-IV)	0
New permanent pacemaker implantation due to procedure related conduction abnormalities	6 (8.5%)
Surgery or intervention related to the device	0

**Table 5:** Hemodynamic performance (echocardiographic assessment at 30 days) of the Myval THV in BAV.

Parameter	
LV EF%	56 ± 10
Transvalvular maximum velocity m/s	1.9 ± 0.5
Transvalvular mean pressure gradient mmHg	9.8 ± 4.5
Effective orifice area cm^2^	1.8 ± 0.4
Indexed effective orifice area cm^2^/m^2^	1.0 ± 0.27
Indexed Stroke volume mL/m^2^	44 ± 17
Dimensionless velocity index	0.58 ± 0.16
Post-TAVI AR	*n* (%)
None/trace	52 (76.5%)
Mild	14 (20.5%)
Moderate	2 (3%)
Severe	0
Patient prosthesis mismatch (PPM)	*n* (%)
BMI < 30 kg/cm^2^	
Moderate PPM (iEOA 0.66 and 0.85 cm^2^/m^2^)	8 (14.8%)
Severe PPM (iEOA ≤ 0.65 cm^2^/m^2^)	3 (5.6%)
BMI ≥ 30 kg/cm^2^ (*n*)	
Moderate PPM (iEOA 0.56 and 0.70 cm^2^/m^2^)	2 (3.7%)
Severe PPM (iEOA ≤ 0.55 cm^2^/m^2^)	0

Data are presented as mean ± SD or number (*n*) and frequency (%).

## Data Availability

Data are contained within the article.

## Supplementary Materials

The following supporting information can be downloaded at: https://www.mdpi.com/article/10.3390/jcm11020443/s1, Table S1: Missing data of the Myval bicuspid registry; Table S2: the collaborating centers and number of patients of the Myval bicuspid registry.

## References

[1] Masri A., Svensson L.G., Griffin B.P., Desai M.Y. (2017). Contemporary natural history of bicuspid aortic valve disease: A systematic review. Heart.

[2] Roberts W.C., Ko J.M. (2005). Frequency by decades of unicuspid, bicuspid, and tricuspid aortic valves in adults having isolated aortic valve replacement for aortic stenosis, with or without associated aortic regurgitation. Circulation.

[3] Vincent F., Ternacle J., Denimal T., Shen M., Redfors B., Delhaye C., Simonato M., Debry N., Verdier B., Shahim B. (2021). Transcatheter Aortic Valve Replacement in Bicuspid Aortic Valve Stenosis. Circulation.

[4] Vahanian A., Beyersdorf F., Praz F., Milojevic M., Baldus S., Bauersachs J., Capodanno D., Conradi L., De Bonis M., De Paulis R. (2021). 2021 ESC/EACTS Guidelines for the management of valvular heart disease. Eur. Heart J..

[5] Otto C.M., Nishimura R.A., Bonow R.O., Carabello B.A., Erwin J.P., Gentile F., Jneid H., Krieger E.V., Mack M., McLeod C. (2021). 2020 ACC/AHA Guideline for the Management of Patients With Valvular Heart Disease: A Report of the American College of Cardiology/American Heart Association Joint Committee on Clinical Practice Guidelines. J. Am. Coll. Cardiol..

[6] Michelena H.I., Della Corte A., Evangelista A., Maleszewski J.J., Edwards W.D., Roman M.J., Devereux R.B., Fernandez B., Asch F.M., Barker A.J. (2021). International Consensus Statement on Nomenclature and Classification of the Congenital Bicuspid Aortic Valve and Its Aortopathy, for Clinical, Surgical, Interventional and Research Purposes. Ann. Thorac. Surg..

[7] Sievers H.H., Schmidtke C. (2007). A classification system for the bicuspid aortic valve from 304 surgical specimens. J. Thorac. Cardiovasc. Surg..

[8] Michelena H.I., Della Corte A., Evangelista A., Maleszewski J.J., Edwards W.D., Roman M.J., Devereux R.B., Fernandez B., Asch F.M., Barker A.J. (2021). International consensus statement on nomenclature and classification of the congenital bicuspid aortic valve and its aortopathy, for clinical, surgical, interventional and research purposes. Eur. J. Cardio-Thorac. Surg..

[9] Kawashima H., Serruys P.W., Mylotte D., Rosseel L., Amat-Santos I.J., Rao R.S., Onuma Y., Wijns W., Abdel-Wahab M., Baumbach A. (2021). Operator preference and determinants of size selection when additional intermediate-size aortic transcatheter heart valves are made available. Int. J. Cardiol..

[10] Garcia-Gomez M., Delgado-Arana J.R., Halim J., De Marco F., Trani C., Martin P., Won-Keun K., Montorfano M., den Heijer P., Bedogni F. (2021). Next-generation balloon-expandable Myval transcatheter heart valve in low-risk aortic stenosis patients. Catheter. Cardiovasc. Interv..

[11] Delgado-Arana J.R., Gordillo-Monge M.X., Halim J., De Marco F., Trani C., Martin P., Infusino F., Ancona M., den Heijer P., Bedogni F. (2021). Early clinical and haemodynamic matched comparison of balloon-expandable valves. Heart.

[12] Sharma S.K., Rao R.S., Chandra P., Goel P.K., Bharadwaj P., Joseph G., Jose J., Mahajan A.U., Mehrotra S., Sengottovelu G. (2020). First-in-human evaluation of a novel balloon-expandable transcatheter heart valve in patients with severe symptomatic native aortic stenosis: The MyVal-1 study. EuroIntervention.

[13] Erbel R., Aboyans V., Boileau C., Bossone E., Bartolomeo R.D., Eggebrecht H., Evangelista A., Falk V., Frank H., Gaemperli O. (2014). 2014 ESC Guidelines on the diagnosis and treatment of aortic diseases: Document covering acute and chronic aortic diseases of the thoracic and abdominal aorta of the adult. The Task Force for the Diagnosis and Treatment of Aortic Diseases of the European Society of Cardiology (ESC). Eur. Heart J..

[14] Varc-3 Writing C., Genereux P., Piazza N., Alu M.C., Nazif T., Hahn R.T., Pibarot P., Bax J.J., Leipsic J.A., Blanke P. (2021). Valve Academic Research Consortium 3: Updated endpoint definitions for aortic valve clinical research. Eur. Heart J..

[15] Kappetein A.P., Head S.J., Genereux P., Piazza N., van Mieghem N.M., Blackstone E.H., Brott T.G., Cohen D.J., Cutlip D.E., van Es G.A. (2012). Updated standardized endpoint definitions for transcatheter aortic valve implantation: The Valve Academic Research Consortium-2 consensus document (VARC-2). Eur. J. Cardio-Thorac. Surg..

[16] Lancellotti P., Pibarot P., Chambers J., Edvardsen T., Delgado V., Dulgheru R., Pepi M., Cosyns B., Dweck M.R., Garbi M. (2016). Recommendations for the imaging assessment of prosthetic heart valves: A report from the European Association of Cardiovascular Imaging endorsed by the Chinese Society of Echocardiography, the Inter-American Society of Echocardiography, and the Brazilian Department of Cardiovascular Imaging. Eur. Heart J. Cardiovasc. Imaging.

[17] Zamorano J.L., Badano L.P., Bruce C., Chan K.L., Goncalves A., Hahn R.T., Keane M.G., La Canna G., Monaghan M.J., Nihoyannopoulos P. (2011). EAE/ASE recommendations for the use of echocardiography in new transcatheter interventions for valvular heart disease. Eur. Heart J..

[18] Zoghbi W.A., Asch F.M., Bruce C., Gillam L.D., Grayburn P.A., Hahn R.T., Inglessis I., Islam A.M., Lerakis S., Little S.H. (2019). Guidelines for the Evaluation of Valvular Regurgitation After Percutaneous Valve Repair or Replacement: A Report from the American Society of Echocardiography Developed in Collaboration with the Society for Cardiovascular Angiography and Interventions, Japanese Society of Echocardiography, and Society for Cardiovascular Magnetic Resonance. J. Am. Soc. Echocardiogr..

[19] Kawashima H., Wang R., Mylotte D., Jagielak D., De Marco F., Ielasi A., Onuma Y., den Heijer P., Terkelsen C.J., Wijns W. (2021). Quantitative Angiographic Assessment of Aortic Regurgitation after Transcatheter Aortic Valve Implantation among Three Balloon-Expandable Valves. Glob. Heart.

[20] Otto C.M., Newby D.E. (2021). Transcatheter Valve Replacement for Bicuspid Aortic Stenosis. JAMA.

[21] Gorla R., Casenghi M., Finotello A., De Marco F., Morganti S., Regazzoli D., Bianchi G., Acerbi E., Popolo Rubbio A., Brambilla N. (2021). Outcome of transcatheter aortic valve replacement in bicuspid aortic valve stenosis with new-generation devices. Interact. Cardiovasc. Thorac. Surg..

[22] Iannopollo G., Romano V., Buzzatti N., Ancona M., Ferri L., Russo F., Bellini B., Granada J.F., Chieffo A., Montorfano M. (2020). Supra-annular sizing of transcatheter aortic valve prostheses in raphe-type bicuspid aortic valve disease: The LIRA method. Int. J. Cardiol..

[23] Yoon S.H., Kim W.K., Dhoble A., Milhorini Pio S., Babaliaros V., Jilaihawi H., Pilgrim T., De Backer O., Bleiziffer S., Vincent F. (2020). Bicuspid Aortic Valve Morphology and Outcomes After Transcatheter Aortic Valve Replacement. J. Am. Coll Cardiol..

[24] Makkar R.R., Yoon S.H., Chakravarty T., Kapadia S.R., Krishnaswamy A., Shah P.B., Kaneko T., Skipper E.R., Rinaldi M., Babaliaros V. (2021). Association Between Transcatheter Aortic Valve Replacement for Bicuspid vs Tricuspid Aortic Stenosis and Mortality or Stroke Among Patients at Low Surgical Risk. JAMA.

[25] Ielasi A., Moscarella E., Mangieri A., Giannini F., Tchetche D., Kim W.K., Sinning J.M., Landes U., Kornowski R., De Backer O. (2021). Procedural and clinical outcomes of type 0 versus type 1 bicuspid aortic valve stenosis undergoing trans-catheter valve replacement with new generation devices: Insight from the BEAT international collaborative registry. Int. J. Cardiol..

[26] Weir-McCall J.R., Attinger-Toller A., Blanke P., Perlman G.Y., Sellers S.L., Wood D., Webb J.G., Leipsic J. (2020). Annular versus supra-annular sizing for transcatheter aortic valve replacement in bicuspid aortic valve disease. J. Cardiovasc. Comput. Tomogr..

[27] Petronio A.S., Angelillis M., De Backer O., Giannini C., Costa G., Fiorina C., Castriota F., Bedogni F., Laborde J.C., Sondergaard L. (2020). Bicuspid aortic valve sizing for transcatheter aortic valve implantation: Development and validation of an algorithm based on multi-slice computed tomography. J. Cardiovasc. Comput. Tomogr..

[28] Kim W.K., Renker M., Rolf A., Fischer-Rasokat U., Wiedemeyer J., Doss M., Mollmann H., Walther T., Nef H., Hamm C.W. (2019). Annular versus supra-annular sizing for TAVI in bicuspid aortic valve stenosis. EuroIntervention.

