# Supplementary material for: Safety and Efficacy of Myval Implantation in Patients with Severe Bicuspid Aortic Valve Stenosis—A Multicenter Real-World Experience
Source: J Clin Med. 2022 Jan 15;11(2):443. doi: 10.3390/jcm11020443 (PMC8779274; doi:10.3390/jcm11020443)
Supplement: Supplementary file 1 [file jcm-11-00443-s001.zip › jcm-1544749-supplementary.pdf]

Journal of
Clinical Medicine

MDPI

Table S1. Missing data of the Myval bicuspid registry.

Data Variable	Missed n (%)
Age	0
Body surface area (BSA)	0
**BAV type (Sievers' classification)**	6 (9%)
STS score	10 (15%)
Effective orifice area (EOA)	18 (26%)
indexed Effective orifice area (iEOA)	15 (22%)
Transvalvular mean pressure gradient	5 (7%)
Indexed stroke volume (iSV)	13 (19%)
Transvalvular maximum velocity	13 (19%)
Dimensionless velocity index (DVI)	17 (25%)
LV ejection fraction	1 (1.5%)
Gender	0
Peripheral vascular disease	0
Prior atrial fibrillation	0
NYHA class	0
Arterial access	1 (1.5%)
Balloon pre-dilatation	0
Balloon post-dilatation	0
Implanted Myval size	0
Conversion to surgery	0
Coronary obstruction	0
Aortic root injury	0
Implantation of the second valve	0
Major vascular complications	0
Life-threatening or disabling bleeding	0
Procedural Death	0
Post TAVI aortic regurgitation	0
Pacemaker implantation	0
Stroke	0
Acute kidney injury stage II or III	0
Aortic valve reintervention	0
30-Day all-cause death	0

Table S2. the collaborating centres and number of patients of the Myval bicuspid registry.

The Centre	The Country	No. of Patients
Christian Medical College & Hospital (CMC) VELLORE	India	12
Aarhus University Hospital (AUH), Aarhus	Denmark	11
Odense University Hospital, Odense	Denmark	11
Apollo Main Hospital, Chennai, India	India	8
Fortis Escorts Heart Institute (FEHI), New Delhi.	India	8
Apollo Hospitals, Apollo Health City, Jubilee Hills, Hyderabad.	India	4
IRCCS Policlinico San Donato, Milan.	Italy	4
King George's Medical University (KGMU) – Lucknow.	India	2
Care Institute of Medical Sciences (CIMS) Hospital, Ahmedabad.	India	2
Asian Heart Institute, Mumbai.	India	2
Istituto Clinico S.Ambrogio, Milan.	Italy	2
University Hospital Dubrava, Zagreb	Croatia	2
